# Acute Myocardial Infarction After Coronavirus Vaccine: A Rare Adverse Effect

**DOI:** 10.7759/cureus.21544

**Published:** 2022-01-24

**Authors:** Sameen Iqbal, Ghufran Adnan, Awais Farhad, Intisar Ahmed, Muhammad Nasir Rahman

**Affiliations:** 1 Cardiology, Aga Khan University, Karachi, PAK

**Keywords:** vaccine, moderna, covid-19, myocardial infarction, acute

## Abstract

A 61-year-old male presented to the emergency department with left arm and jaw pain for three hours which started 90 minutes after receiving the first dose of Moderna vaccine for coronavirus disease 2019 (COVID-19). He had a prior history of ischemic heart disease. Initial investigations confirmed the diagnosis of acute coronary syndrome. The patient was managed for non-ST-elevation myocardial infarction and percutaneous coronary intervention to the right posterior descending artery was done, and he was discharged after two days of hospital stay. As the patient was doing well for many years and was compliant with medications, this event was likely triggered by the coronavirus vaccine. Healthcare providers should be aware of the side effects of the vaccine and further investigations should be carried out in high-risk patients before vaccination. However, worldwide coronavirus vaccination programs play a significant role to halt this pandemic and these rare adverse side effects of the vaccine should never discourage people from the vaccination but monitoring of evolving data by the concerned authorities is very important so that these events can be prevented in future.

## Introduction

Moderna coronavirus disease 2019 (COVID-19) vaccine (mRNA-1273 vaccine) was issued an emergency use authorization (EUA) by the U.S. Food and Drug Administration on December 18, 2020, for the prevention of coronavirus disease 2019 (COVID-19) caused by severe acute respiratory syndrome coronavirus 2 (SARS-CoV-2). The mRNA-1273 vaccine showed 94.1% efficacy at preventing COVID-19 illness, including severe disease. The safety of the Moderna vaccine has been excellent with the majority of the events that are related to local and systemic reactions in the clinical trial data [1]. However, some cases of anaphylaxis, thromboembolism, and cardiac events have been reported outside of the clinical trials [2-4]. We report a case of a 61-year-old male with a prior history of ischemic heart disease, presenting with non-ST-elevation myocardial infarction after having the first dose of Moderna vaccine. However, the myocardial infarction in our case was caused by the vaccine is not ascertained.

## Case presentation

A 61-year-old male with a history of diabetes, hypertension, and ischemic heart disease came to the emergency room (ER) with complaints of the left arm and jaw pain for a duration of three hours which started about 90 minutes after having the first dose of Moderna COVID-19 vaccine in the right arm. He had acute coronary syndrome eight years back and had undergone angioplasty of mid-segment of the right coronary artery (RCA); he has been doing well over the period of the last eight years. At that time, he had a mild disease in the left anterior descending artery and the rest of the vessels were free of disease. The patient had been compliant with medical therapy and his risk factors were well controlled.

**Figure 1:**
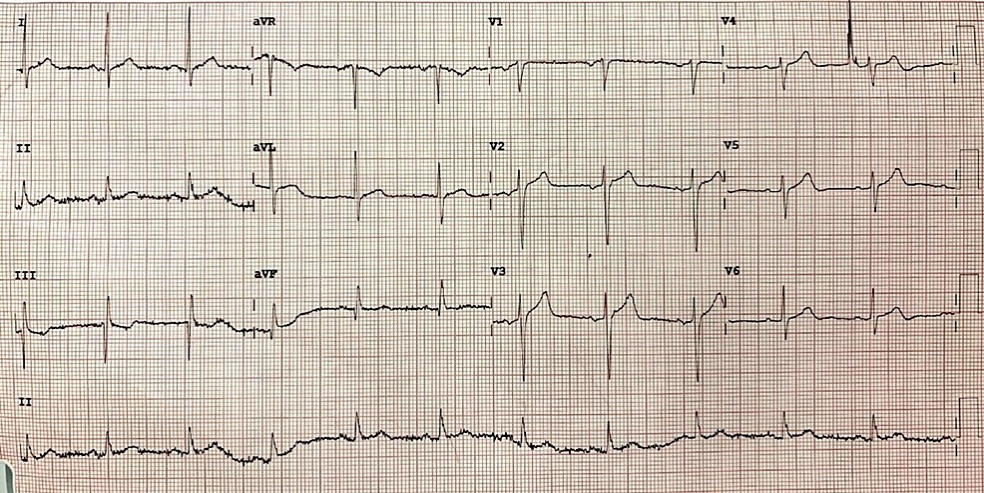
Twelve-lead electrocardiogram on arrival in the emergency room shows normal sinus rhythm with no ST/T changes suggestive of ischemia. aVR: augmented vector right; aVL: augmented vector left; aVF: augmented vector foot

On arrival to the ER, his pulse was 64 beats/minute, blood pressure was 134/82 mmHg, respiratory rate was 16 breaths/minute, and pulse oximetry (SpO_2_) measured 99% on room air. The rest of the physical examination was unremarkable. He had no other symptoms including shortness of breath, palpitations, or diaphoresis. He was compliant with medications and was not having any symptoms previously. An electrocardiogram (ECG) was done which showed normal sinus rhythm with no ST-T changes suggesting ischemia (Figure 1).

All laboratory tests including complete blood count, blood chemistry containing blood urea nitrogen, creatinine, and electrolytes were unremarkable except cardiac enzymes which were significantly raised with initial high-sensitivity troponin-I of 77ng/L (normal value: < 57ng/L) and a peak of 754 ng/L. COVID-19 polymerase chain reaction (PCR) for screening was also done which came out to be negative. An echocardiogram was done which showed normal left ventricular systolic function with no wall motion abnormalities and normal functioning cardiac valves.

**Figure 2:**
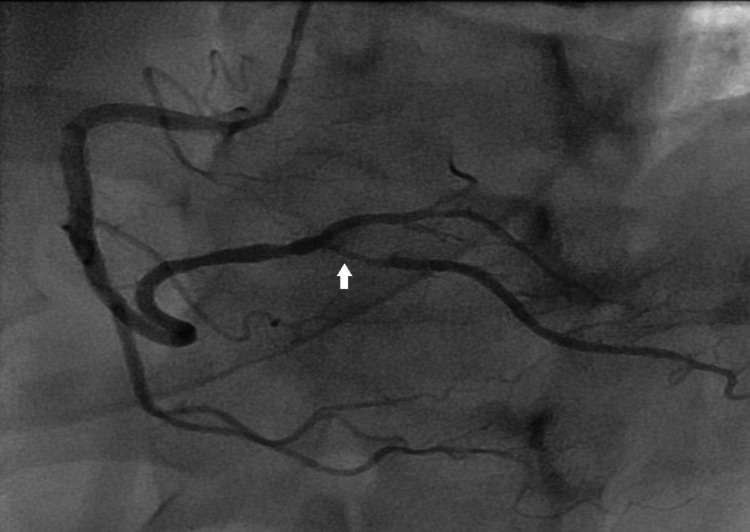
Initial angiogram of RCA shows the patent stent in the mid-segment of RCA and severe disease in ostioproximal segment of the right posterior descending artery (arrow). RCA: right coronary artery

Following the laboratory workup which was coherent with non-ST elevation myocardial infarction (NSTEMI) with thrombolysis in myocardial infarction (TIMI) score of 3, left heart catheterization and revascularization was planned the next day. A coronary angiogram was performed via a right radial artery which showed a patent right coronary artery (RCA) stent in the mid-segment with severe disease in the ostioproximal segment of the right posterior descending artery (RPDA) likely due to the plaque rupture leading to myocardial infarction (Figure 2).

**Figure 3:**
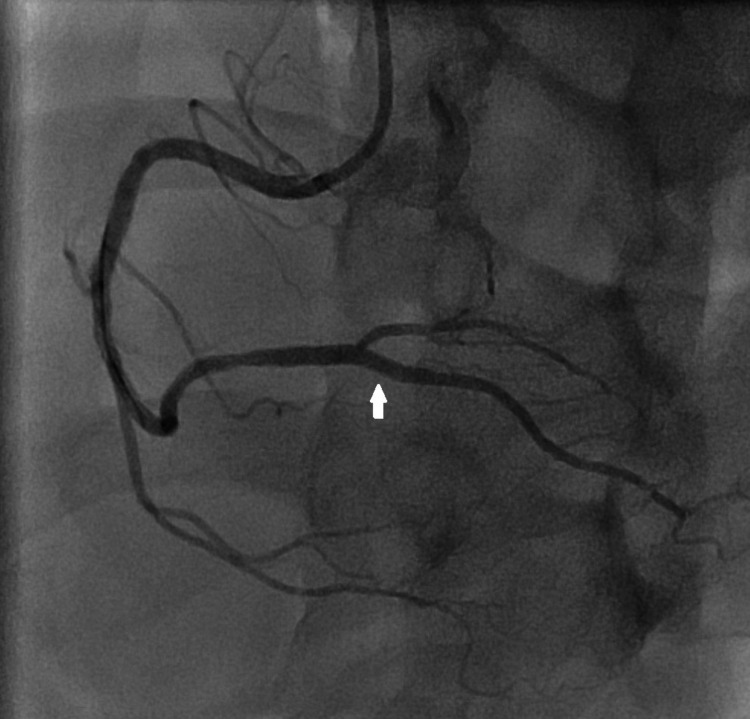
RCA angiogram after percutaneous coronary intervention shows optimally expanded ostioproximal segment of right posterior descending artery (arrow). RCA: right coronary artery

There was also mild disease in the mid-segment of the left anterior descending artery (LAD) which was similar to the last angiogram performed eight years ago. He was given aspirin, clopidogrel, and intravenous anticoagulation before left heart catheterization. The percutaneous coronary intervention was performed on the RPDA. The culprit lesion of RPDA was wired with workhorse Sion 0.014 wire (Tustin, CA: Asahi Intecc USA, Inc.) and the right posterolateral ventricular branch (RPLV) was wired with workhorse BMW 0.014 wire (Chicago, IL: Abbott Laboratories). The culprit lesion was predilated with a semi-compliant balloon with 2.0 × 10 mm at 12 atmospheres. Everolimus eluting stent 2.5 × 20 mm deployed in distal RCA to RPDA at 8 atmospheres. The proximal segment in the distal RCA was post dilated with non-compliant 3.0 × 8 mm at 10 atmospheres while the RPDA segment was post dilated with non-compliant 2.5 × 8 mm at 16-18 atmosphere. The final angiogram showed excellent results with TIMI 3 flow (Figure 3).

The patient remained stable postprocedure and his symptoms were improved. Medications including dual antiplatelets, statin, and beta-blockers were optimized. The patient’s hospital course was uneventful and he was discharged after 24 hours. He returned to the clinic one week after discharge and was doing well.

## Discussion

Moderna (mRNA -1234) is a lipid-nanoparticle (LNP)-encapsulated mRNA vaccine that expresses the perfusion-stabilized spike glycoprotein of severe acute respiratory syndrome coronavirus 2 (SARS-CoV-2) [5]. It has the potential to elicit a highly S protein-specific antiviral response [5]. It was approved by the U.S. Food and Drug Administration for emergency use on December 18, 2020, and in the United Kingdom, it was approved on January 8, 2021, for the prevention of COVID-19 caused by SARS-CoV-2.

The coronavirus efficacy (COVE) phase three trial showed 94.1% efficacy at preventing COVID-19 illness, including severe disease [1]. According to the Vaccine Adverse Event Reporting System (VAERS) and V-safe system of the US Centers for Disease Control and Prevention, the rates of non-serious adverse effects following immunization (AEFI) after public administration of mRNA-1273 was similar to the clinical trials. Local reactions including injection-site pain and tenderness, and fatigue and headache were among the most common systemic reactions reported [6]. However, few cases of venous thromboembolism and myocardial infarction have been described in literature after the Moderna vaccine [3-4,7]. Recently, myocarditis and pericarditis have also been reported after mRNA vaccines, with myocarditis mainly in younger male individuals and pericarditis in old-aged patients [8].

Cardiovascular diseases cause approximately one-third of deaths worldwide. Among cardiovascular illnesses, ischemic heart disease (IHD) is the most prevalent. The primary pathological process that leads to IHD is atherosclerosis, an inflammatory disease of the arteries associated with lipid deposition and metabolic alterations due to multiple risk factors [9]. However, the association of myocardial infarction and COVID-19 vaccination is not a usual entity, it has been reported in the literature. Boivin and Martin described the case of a 96-year-old female having myocardial infarction in few hours after the first dose of Moderna vaccine but the patient declined cardiac catheterization and was medically managed [7]. Sung et al. also reported a case series of two patients with acute myocardial infarction within 24 hours of the mRNA-1273 vaccine with involvement of the left circumflex artery on cardiac catheterization [4]. Kumar et al. stated a case series of two patients who had similar events after the first dose of the Covishield vaccine with one patient having thrombotic occlusion of mid-RCA and the other patient was given streptokinase for anterior wall myocardial infarction (MI) and later drug-eluting stent was placed in left anterior descending artery [10].

Here, we presented the case of a 61-year-old man who had a myocardial infarction within 24 hours after the first dose of Moderna vaccine with possible plaque rupture as the cause of MI in our case. Patients presenting with cardiac symptoms to the emergency room post-vaccination should be evaluated for the possibility of acute coronary syndromes as timely management plays an important role in the outcomes of these patients. Additionally, as a precautionary measure, healthcare providers should consider additional screenings for older adults before COVID-19 vaccine administration if there are rising cases of these adverse events in the adult population [8].

The benefits of COVID-19 vaccination still outweigh these rare serious side effects and it is the only way to fight this pandemic which has taken millions of lives globally. Nevertheless, the awareness of such vaccine-related adverse events is essential for the medical community.

## Conclusions

Reporting of the serious side effects following COVID-19 vaccination is important for creating awareness among the scientific community. Patients presenting with cardiac symptoms after vaccination should be evaluated for acute coronary syndromes as early identification of such symptoms plays a significant role in the management of such patients. Healthcare providers should be cognizant of the rare adverse effects of the COVID-19 vaccines so that necessary actions needed to be taken before giving the vaccination, especially to the older patients with multiple comorbidities. The only way to curtail the COVID-19 pandemic is through mass vaccination programs and people should not be discouraged by the rare side effects of the vaccine as the benefits outweigh the risks.
